# Evaluation of Five Candidate Genes from GWAS for Association with Oligozoospermia in a Han Chinese Population

**DOI:** 10.1371/journal.pone.0080374

**Published:** 2013-11-26

**Authors:** Miaofei Xu, Yufeng Qin, Jianhua Qu, Chuncheng Lu, Ying Wang, Wei Wu, Ling Song, Shoulin Wang, Feng Chen, Hongbing Shen, Jiahao Sha, Zhibin Hu, Yankai Xia, Xinru Wang

**Affiliations:** 1 State Key Laboratory of Reproductive Medicine, Institute of Toxicology, Nanjing Medical University, Nanjing, China; 2 Key Laboratory of Modern Toxicology of Ministry of Education, School of Public Health, Nanjing Medical University, Nanjing, China; 3 School of Public Health, Nantong University, Nantong, China; 4 Department of Epidemiology and Biostatistics and Key Laboratory of Modern Toxicology of Ministry of Education, School of Public Health, Nanjing Medical University, Nanjing, China; MOE Key Laboratory of Environment and Health, School of Public Health, Tongji Medical College, Huazhong University of Science and Technology, China

## Abstract

**Background:**

Oligozoospermia is one of the severe forms of idiopathic male infertility. However, its pathology is largely unknown, and few genetic factors have been defined. Our previous genome-wide association study (GWAS) has identified four risk loci for non-obstructive azoospermia (NOA).

**Objective:**

To investigate the potentially functional genetic variants (including not only common variants, but also less-common and rare variants) of these loci on spermatogenic impairment, especially oligozoospermia.

**Design, Setting, and Participants:**

A total of 784 individuals with oligozoospermia and 592 healthy controls were recruited to this study from March 2004 and January 2011.

**Measurements:**

We conducted a two-stage study to explore the association between oligozoospermia and new makers near NOA risk loci. In the first stage, we used next generation sequencing (NGS) in 96 oligozoospermia cases and 96 healthy controls to screen oligozoospermia-susceptible genetic variants. Next, we validated these variants in a large cohort containing 688 cases and 496 controls by SNPscan for high-throughput Single Nucleotide Polymorphism (SNP) genotyping.

**Results and Limitations:**

Totally, we observed seven oligozoospermia associated variants (rs3791185 and rs2232015 in *PRMT6*, rs146039840 and rs11046992 in *Sox5*, rs1129332 in *PEX10*, rs3197744 in *SIRPA*, rs1048055 in *SIRPG*) in the first stage. In the validation stage, rs3197744 in *SIRPA* and rs11046992 in *Sox5* were associated with increased risk of oligozoospermia with an odds ratio (OR) of 4.62 (*P*  =  0.005, 95%CI 1.58-13.4) and 1.82 (*P*  =  0.005, 95%CI 1.01-1.64), respectively. Further investigation in larger populations and functional characterizations are needed to validate our findings.

**Conclusions:**

Our study provides evidence of independent oligozoospermia risk alleles driven by variants in the potentially functional regions of genes discovered by GWAS. Our findings suggest that integrating sequence data with large-scale genotyping will serve as an effective strategy for discovering risk alleles in the future.

## Introduction

Infertility is a common reproductive disease and male factor infertility accounts for half of this problem [1], [2]. Genetic causes are responsible for 10–15% of severe male infertility, including chromosome number defects, Y chromosome microdeletions and autosomal chromosome mutations [3], [4]. Although enormous progress in the understanding of human reproduction, about 50% cases are still defined as idiopathic infertility because of the unknown causes [5].

A significant proportion of idiopathic male infertility is accompanied by abnormal semen quality, mainly oligozoospermia. Genetic variants of genes involved in spermatogenesis may be associated with spermatogenic impairment [6]. Identification of potentially functional genetic variation in spermatogenesis will improve our understanding of idiopathic infertility etiology and will contribute to the development of targeted therapies. However, to date, only a few genetic variants have been identified to be associated with oligozoospermia.

More recently, genome-wide association studies (GWAS) investigated idiopathic male infertility [7], but the interpretation of the results was limited by the small sample capacity. Our subsequent GWAS with larger sample size identified four susceptibility loci associated with non-obstructive azoospermia (NOA) in Han Chinese [8]. And implicated genes for the four susceptibility loci were *PRMT6* (*protein arginine methyltransferase 6*), *PEX10* (*peroxisomal biogenesis factor 10*), *SOX5* (*SRY-related HMG-box gene 5*), *SIRPA* (*signal regulatory protein, α-1*) and *SIRPG* (*signal regulatory protein, β-2*). Although these loci showed evidence for association with NOA in Han Chinese men, it is unknown whether they also contribute to the susceptibility of oligozoospermia.

Rapid technological advances in next generation sequencing (NGS) have opened the door to discover all possible genetic variations in the entire genome including not only common alleles detected by GWAS, but also rare, causal variants. In order to investigate potentially causal variants of the candidate genes on oligozoospermia, we carried out a two-stage study by using NGS in the discovery phase and SNPscan in the follow-up validation phase.

## Materials and Methods

### Study subjects

The study was approved by the Ethics Review Board of Nanjing Medical University. The protocol and consent form were approved by the Institutional Review Board of Nanjing Medical University prior to the study. All participants provided their written informed consent to join in this study. We performed a two-step case-control analysis. The first stage included 96 idiopathic male infertility with oligozoospermia and 96 healthy controls. 688 oligozoospermia cases and 496 controls were recruited in the second stage. Some cohorts within the sample sets have been reported in previously published data [9], [10]. The patients recruited from the Center of Reproductive Medicine between March 2004 and January 2011, were diagnosed as infertility without infertile wives. Patients were selected on the basis of a comprehensive andrological examination, including medical history and physical examination, hormone analysis, karyotype, and Y chromosome microdeletion screening. Patients with a history of orchitis, obstruction of vas deferens, chromosomal abnormalities, or microdeletions of azoospermia factor (*AZF*) region on the Y chromosome were excluded. All controls with normal reproductive function were from the early pregnancy registry of the same hospitals, whose wives were in the first trimester of pregnancy and confirmed as having healthy babies 6–8 months later. After completing a questionnaire, each subject donated 5 ml of blood which was used for genomic DNA extraction and an ejaculate for semen analysis. Semen analysis for sperm concentration and motility was performed following World Health Organization criteria [11].

### Solexa sequencing

The exons and promoters of genes were amplified using polymerase chain reaction (PCR) in 48 overlapping fragments, by the use of the primer pairs shown in Table S1. After PCR, by the use of DNase I (Fermentas Life sciences), a fragmented DNA sequences library of each participant was created and purified using the QIA quick purification kit (QIAGEN). After the step of DNA End-Repair and A-Tailing, the total DNA was ligated to the PE Adapter oligo mix with T4 DNA Ligase and then followed by 2% TBE PAGE gel purification with size selection. The purified DNA was used directly for cluster generation and sequencing analysis using the Illumina Solexa Sequencer according to the manufacturer’s instructions. After performing the image analysis and base calling, we received the primary data in FASTQ format. The subsequent procedures performed with Solexa were summarizing data production, evaluating sequencing quality, calculating length distribution of reads and filtrating reads contaminated. To identify single-nucleotide variants (SNVs) and indels, clean reads were aligned against hg19. SNVs and indels were identified using Samtools.

### Follow-up genotyping by SNPscan sequencing

Selected SNPs were genotyped by a custom-by-design 48-Plex SNPscan™ Kit (Cat#:G0104; Genesky Biotechnologies Inc., Shanghai, China). This kit was developed according to patented SNP genotyping technology by Genesky Biotechnologies Inc., which was based on double ligation and multiplex fluorescence PCR [12]. In order to validate the genotyping accuracy using SNPscan™ Kit, 5% duplicate samples were analyzed by single nucleotide extension using the Multiplex SNaPshot Kit (Applied Biosystems Inc., Foster City, CA, USA), and the concordance rates were more than 99%.

### Statistical analysis

Statistical analysis was performed using PLINK (version 1.07; http://pngu.mgh.harvard.edu/~purcell/pli∼nk/). and Stata 10.0 (Stata Corp LP, USA). Hardy-Weinberg equilibrium (HWE) tests were performed on online software, SHEsis (http://analysis.bio-x.cn/myAnalysis.php). Infertility risks were estimated with odds ratios (OR) and 95% confidence intervals (95%CI) using multivariate logistic regression. Bonferroni adjustment for multiple testing was applied. *P*-value for a truly significant result was calculated as 0.05/n.

## Results

### Identification of coding variants in biological candidate genes

By using NGS, we identified a total of 287 genetic variations in our candidate genes in 96 cases with oligozoospermia and 96 healthy controls. Because of the exploratory nature of the analysis, *P*-value < 0.2 was considered statistically suggestive. In this study, we used genotypic and other association models to obtain the minimum *P*-value, and identified 59 out of 287 genetic variations, among which, seven genetic variants (rs1129332 in *PEX10*, rs3791185 and rs2232015 in *PRMT6*, rs3197744 in *SIRPA*, rs1048055 in *SIRPG*, rs146039840 and rs11046992 in *Sox5*) were predicted to be potentially functional and chosen to be replicated in the follow-up study. The genotype distribution of the selected variants were presented in Table 1.

**Table 1:** Number of Coding Variants Identified in the Biological Candidate Genes through Exon Sequencing.

Gene	SNP ID	Region	Position	Nucleotide change	Position	*P* a
***PEX10***	rs1129332	1p36.32	2336210	C/T	downstream, 3’-UTR	0.158
***PRMT6***	rs3791185	1p13.3	107600869	G/A	3’-UTR	0.118
	rs2232015	1p13.3	107599258	A/T	upstream	0.161
***SIRPA***	rs3197744	20p13	1918487	G/T	3’-UTR	0.155
***SIRPG***	rs1048055	20p13	1610062	A/C	3’-UTR	0.179
***SOX5***	rs146039840	12p12.1	24102616	T/G	5’-UTR	0.023
	rs11046992	12p12.1	23737566	G/A	upstream	0.090

a The minimum *P*-value was obtained from different genetic association models.

### Associations between oligozoospermia-predisposed variants and spermatogenic impairment

In the second stage, we replicated these seven variants in 496 controls and 688 cases. Their associations with oligozoospermia were shown in Table 2. Most SNPs were common (MAF>5%), only rs146039840 in *SOX5* was rare (MAF≤2%). Among these variants, rs3197744 (G>T) and rs11046992 (G>A) were associated with oligozoospermia. The TT genotype of rs3197744 in the 3’-UTR region of *SIRPA* increased the risk of oligozoospermia with OR of 4.62 (1.58–13.47), compared with the GG genotype. And the genotype frequencies of rs11046992 in *SOX5* were 41.13% (GG), 46.66% (GA) and 12.06% (AA) in the cases and 48.79% (GG), 43.15% (GA) and 7.86% (AA) in the controls. Logistic regression analysis revealed that rs11046992 AA genotype was associated with a significantly increased risk of oligozoospermia, compared with the GG genotype (*P*  =  0.005, OR  =  1.82(1.20–2.76)).

**Table 2:** Association of seven identified genetic variations in solexa sequence with oligozoospermia in a Chinese population.

Gene	SNP	Chr	Position	Genotype	Case	Control	MAF		OR^a^ (95%CI)	*P* a
							Case	Control		
***PEX10***	rs1129332	1p36.32	2336210	C/C	219	150	0.44	0.46	1.00	
				C/T	331	229			0.99(0.76–1.29)	0.941
				T/T	132	108			0.84(0.60–1.16)	0.289
				C/T+T/T	463	337			0.94 (0.73–1.21)	0.634
***PRMT6***	rs3791185	1p13.3	107600869	C/C	514	356	0.14	0.15	1.00	
				C/T	162	129			0.87(0.67–1.14)	0.307
				T/T	12	11			0.76(0.33–1.73)	0.508
				C/T+T/T	174	140			0.86(0.66–1.12)	0.259
	rs2232015	1p13.3	107599258	A/A	353	285	0.27	0.26	1.00	
				A/T	287	167			1.39(1.08–1.78)	0.009
				T/T	44	44			0.81(0.52–1.26)	0.347
				A/T+T/T	331	211			1.27(1.00–1.60)	0.046
***SIRPA***	rs3197744	20p13	1918487	G/G	436	350	0.20	0.15	1.00	
				G/T	228	141			1.30(1.01–1.67)	0.043
				T/T	23	4			4.62(1.58–13.47)	0.005b
				G/T+T/T	251	145			1.39(1.08–1.78)	0.009
***SIRPG***	rs1048055	20p13	1610062	C/C	443	312	0.19	0.20	1.00	
				C/A	212	161			0.93(0.72–1.19)	0.556
				A/A	21	15			0.99(0.50–1.94)	0.968
				C/A+A/A	233	176			0.92(0.73–1.19)	0.573
***SOX5***	rs11046992	12p12.1	23737566	G/G	283	242	0.35	0.29	1.00	
				G/A	321	214			1.28(1.01–1.64)	0.045
				A/A	83	39			1.82(1.20–2.76)	0.005b
				G/A+A/A	404	253			1.37(1.08–1.72)	0.008
	rs146039840	12p12.1	24102616	T/T	674	487	0.007	0.009	1.00	
				T/G	8	9			0.64(0.25–1.68)	0.366
				G/G	1	0				
				T/G+G/G	9	9			0.72(0.28–1.83)	0.492

MAF, minor allele frequency;

a ORs and *P* were obtained from multivariate logistic regression analysis;

b *P*-value was survived after Bonferroni correction.

As to the other SNPs, no significant differences of distribution frequencies were identified between the case and control groups.

## Discussion

Whether NOA associated genes identified in our previous GWAS study contributing to oligozoospermia were still unknown. Thus, we addressed this issue by deep exon-sequencing and large-scale genotyping across five genes discovered by GWAS. In the first discovery stage, we identified seven potentially functional genetic variants, and in the second stage, we validated the associations of *SIRPA*-rs3197744 (G>T) and *Sox5*-rs11046992 (G>A) with the risk of oligozoospermia.

**Figure 1:**
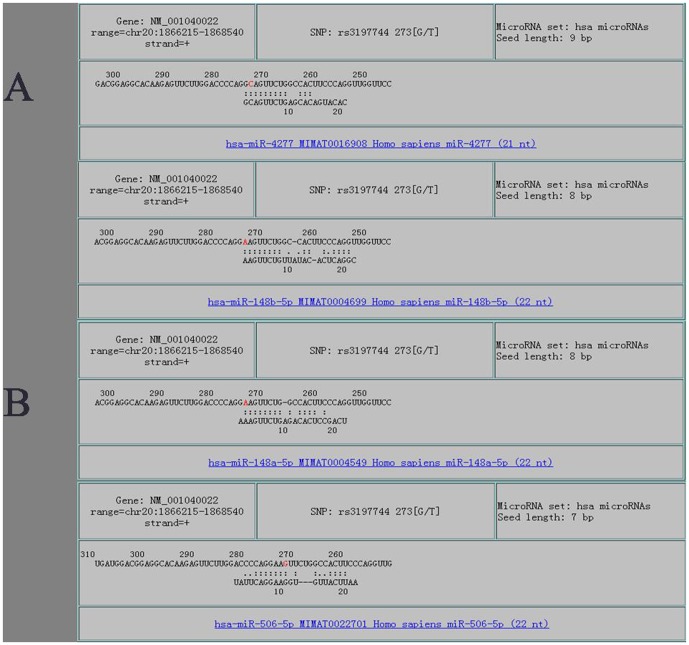
Prediction of the binding of microRNA to the 3’ UTR of *SIRPA* with the substitution of rs3197744 G>T. (A) the original binding of -miR-4277 to the 3’ UTR of *SIRPA* (B) the substitution of rs3197744 G>T may increase the binding sites of miR-148b-5p, miR-148a-5p, miR-506-5p etc.

*SIRPA*, which belongs to the signal regulatory family, is a membrane glycoprotein belonging to the immunoglobulin (Ig) superfamily [13], [14], and is especially abundant in macrophages, dendritic cells, neutrophils, and neurons [15], [16], [17], [18]. Growth factor receptors and growth hormone receptor signaling is suppressed by the up regulation of *SIRPA* [14], [19], [20]. *SIRPA* also regulates the NFkB activity that renders the cells resistant to TNF mediated apoptosis [21]. It was reported that polymorphisms in *SIRPA* modulate engraftment of human hematopoietic stem cells [22]; however, a related role in oligozoospermia has been documented. In this study, we found the rs3197744, which is located in the 3’ UTR region, significantly increased the risk of spermatogenic impairment (*P*  =  0.005). It is believed that microRNAs down-regulated gene expression by the mRNA cleavage or translational repression through base pairing in the 3’ UTR of messenger RNAs (mRNAs) of target genes. Rs3197744 may lead to altered binding activity to microRNAs, which might regulate the gene expression [23]. We used the MicroSNiPer to predict the effects of this SNP on putative microRNA targets [24], [25]. As shown in Fig. 1, we found that rs3197744 substitution may disrupt the binding of miR-4277 to the 3’ UTR of *SIRPA*, and may increase the binding of miR-148a, miR-148b and miR-506.These alternations may change the expression level of *SIRPA* and hence this modifies the susceptibility to oligozoospermia.

*SOX5* is a member of the *SOXD* gene family, which includes three genes, *SOX5*, *SOX6*, and *SOX13* [26]. SOX proteins are transcription factors with a high motility group box DNA binding domain similar to that of the sex-determining region (SRY) protein [27], [28]. The *SOX5* gene encodes two major proteins, the full-length 84-kDa SOX5 (L-SOX5) and the 48-kDa SOX5 (S-SOX5). The S-SOX5 protein is expressed in tissues with motile cilia, suggesting a role of this transcription factor in motile cilia genesis [29]. The *ZNF230* gene, which could be induced by *SOX5,* is a recently cloned gene which is transcribed only in fertile male testis and may be related to human spermatogenesis [30]. In this study, we found the rs11046992 located in the upstream region of *SOX5* significantly increased the risk of spermatogenic impairment (*p* =  0.005). It may affect the binding sites of transcription factors, but the exact molecular mechanisms are unknown.

In conclusion, our two-stage genetic association study provided convincing evidence that two SNPs in the five previously GWAS-identified genes were associated with risk of oligozoospermia. These findings may be useful in understanding male infertility etiology and more epidemiological and functional studies are still needed to validate our findings.

## Supporting Information

Table S1 The forward (F) and reverse (R) primers for multiplex competitive amplification.
(DOCX)
