# Supplementary material for: Evaluation of Five Candidate Genes from GWAS for Association with Oligozoospermia in a Han Chinese Population
Source: PLoS One. 2013 Nov 26;8(11):e80374. doi: 10.1371/journal.pone.0080374 (PMC3841155; doi:10.1371/journal.pone.0080374)
Supplement: Table S1 — The forward (F) and reverse (R) primers for multiplex competitive amplification. (DOCX) [file pone.0080374.s001.docx]

**Table S1.**The forward (F) and reverse (R) primers for multiplex competitive amplification.

| **Gene** | **Location** | **Forward Primer (F)** | **Reverse Primers(R)** | **Amplicon size (bp)** |
| --- | --- | --- | --- | --- |
| ***PEX10*** |  |  |  |  |
| PEX10_1A | chr1:2343998-2345276 | CTCTGGCTTTGTGACTGGGCTGTG | GGCGAGAACTGATGACGGCACGA | 1,279 |
| PEX10_1B | chr1:2343096-2344422 | ACCCAAGCGAAAGCCTCCGTCTC | GCCTCCTGAAGAGCTGGGATTACAGA | 1,327 |
| PEX10_2 | chr1:2341598-2342113 | CACAGGTGGGAGATTCTTCGCAGACT | CCCAGGGACACACAAAGGCTGGAA | 516 |
| PEX10_3 | chr1:2339841-2340340 | GGCAGCAGTCTCAGGGTCCACATAC | GGGTGACAAGGACGGCCTAAGCAG | 500 |
| PEX10_4A | chr1:2337062-2338524 | CTACGAAGCAGGCACCTCAGACTCC | CAGGGTGGCTAGGTTAGTATCTTACATGACAA | 1,463 |
| PEX10_4B | chr1:2336036-2337188 | GCCTGGACACAGATGACCTCTACGG | TCTCTGGCAAGCTGACCTTGACTAACC | 1,153 |
| PEX10_5 | chr1:2334302-2336362 | GCCTATTTCCCGCCTCCATTTGATGT | GGCTGTGGAAGGTCTTGCTGTGTT | 2,061 |
| ***PRMT6*** |  |  |  |  |
| PRMT6_A01 | chr1:107598682+107599253 | CGGTGGCTCACGCTTGTAATCCCA | CGCGCTCGTCCTTCCTACTTCCT | 572 |
| PRMT6_A02 | chr1:107599147+107599830 | GCCAGGACACCAGAAGAGCAATACAA | ACTCGTGCAGGAGTCCGTAGCC | 684 |
| PRMT6_A03 | chr1:107599784+107600447 | GGATGCCATCGTGAGCGAGTGGAT | GTCTTTGGTCTTCTCCTCCTGGTCTCC | 664 |
| PRMT6B | chr1:107600335+107602033 | AAGACACGGACGTTTCAGGAGAGATCA | GGTGACGGTTACAACTGTACTGCTTGCTA | 1,699 |
| ***SIRPA*** |  |  |  |  |
| SIRPA_1A01 | chr20:1874681+1875254 | GCGGCACCGAAGTCCTTCCTACAC | CCGACAGCGTGCCCGTTCAGAA | 574 |
| SIRPA_1A02 | chr20:1875129+1875815 | CTTCCAGTGCCTTCCAGCCCTC | AGAGAGAAACAGACCCAAGGGAGCC | 687 |
| SIRPA_1A03 | chr20:1875764+1876362 | CCTCTCCGTGTCTCTGGCTCTCTG | CCTTGGCTGTGTGACCTTGGGCAT | 599 |
| SIRPA_1_B | chr20:1876248+1877202 | CCTTCGGCTAATGCCGAGCAGTAATAG | ACCCCACTGCCTACAACACAGTTTCAA | 955 |
| SIRPA_2 | chr20:1901951+1903469 | TGTCTCAGAAGGTTCTTAACGTGTCA | CAGTGGTGACTCTTGGAGTGTGG | 1,519 |
| SIRPA_3 | chr20:1905230+1905742 | GAGTCTCGTGGGCAAGTGTGTA | GGCAGTGATGAACATCTTGTAACCA | 513 |
| SIRPA_4 | chr20:1908311+1908811 | AGCAGGACCATCTAGCTTACTCC | CAGTCCACATGGGATTAGACAAGG | 501 |
| SIRPA_4_5 | chr20:1895691+1896201 | AACACAGAGGATCACGTAAGGATGA | GCACCTACCACCACACCTGAT | 511 |
| SIRPA_5 | chr20:1914985+1915709 | TGAGGACATCTTCCAAGCTCTTGA | GAAGGCACGATCCACACTAAGG | 725 |
| SIRPA_6 | chr20:1917868+1920582 | CACAGAAGCATCCAGACTTGGTATT | GGACGCACTCACAAAGACAAGAATA | 2,715 |
| ***SIRPG*** |  |  |  |  |
| SIRPG_1 | chr20:1638088-1638596 | TTCTGCTAACCCTGTCTGATTATCCT | CACAAGGTTTGATGCCTCTGGTAG | 509 |
| SIRPG_2 | chr20:1629618-1630144 | TGCACCCAGTCACTGAATATCTTTG | TGTTGCTCAAAGAATGGAAGGTGTT | 527 |
| SIRPG_3 | chr20:1615763-1617212 | ACTCATCGTTAGTGATTGTCATCTCAG | GACTCTTCGCATGTGGGATTTGG | 1,450 |
| SIRPG_4 | chr20:1609755-1610975 | CCCTCGTCTTCCCTGATTTCCA | TTCTTTGTTTCTGAGTGTGAGGAGTAG | 1,221 |
| ***SOX5*** |  |  |  |  |
| SOX5_1A01 | chr12:24715492-24716049 | CGGGAAGAAGTTGACAAAGGGACTTGA | GTGTTGCTCTCAAGGACGAGGTGAAATC | 558 |
| SOX5_1A02 | chr12:24715079-24715579 | TTGTTATTTGCACTCGCATTGTGTTGTTTG | CCCTCCCTCTGTACTTGAGGAAACTCC | 501 |
| SOX5_1A03 | chr12:24714677-24715229 | CCGATGTTGTTGTGGGCAGACCTC | GGGAGAAGGATTGCAGAAAGCGAAACA | 553 |
| SOX5_1B | chr12:24713688-24714887 | GGAGGTGGTTGATGCGAGAGGAGA | TTTAAAGCCATCTGGCCCTTAACCTAATCA | 1,200 |
| SOX5_2 | chr12:24560185-24560693 | TCACTCCGTATCTGTCTGGTTGG | CACAAGTACTGACAAGAGATGGTAAGA | 509 |
| SOX5_3 | chr12:24521256-24521792 | TTAGAAGCAATTATTCTCAAGAATGGAGTT | TTTAATCTGCCCTATTACTACAGAATCTACA | 537 |
| SOX5_4 | chr12:24429711-24430286 | gAAACAATGCACTGTTAATGGTCTACT | AGAATCATACCCTGGAATACTTAATAGCC | 575 |
| SOX5_5 | chr12:24102315-24102814 | AGACTCCGCTGCTGGAAGTG | GCAAATCAGTTCAGTTTGGTCCG | 500 |
| SOX5_6 | chr12:24048602-24049101 | GCTTTGCAGTGGTATTTGGATGGT | GGTGAATCTTGTGCATGGGTTCTTA | 500 |
| SOX5_7 | chr12:23998642-23999175 | TCTCTCTTTCAGGTAAACAATTATTTGTCA | CTCTGCTGCCACATTCTATTCTTGA | 534 |
| SOX5_8 | chr12:23908378-23908878 | AGTATGGCTTTGTTCCGCAAGAG | TCCATAAGGCACAAACAAAGAAAGA | 501 |
| SOX5_9 | chr12:23893692-23894191 | CTATTGTGCCAGAGAGCCAAGTTC | GGAGGTGGAAGGGACTCTTATTCAT | 500 |
| SOX5_10 | chr12:23887410-23887931 | TTGATCACTGCCGTGGTATCTTAGG | TTGCTTAGATTGAAGCTCCCTGACA | 522 |
| SOX5_11 | chr12:23818176-23818677 | TAGTTCTGCTGTTTAAGGTAGTCCCA | AAGAATATTGGCTTCAGAAGCACAGAT | 502 |
| SOX5_12 | chr12:23793533-23794053 | AGGATGAGGTTTCCGCCTTAATTG | GAAATACGATAAGCTATGTTTAGTGCAG | 521 |
| SOX5_13 | chr12:23757177-23757676 | GGTGAGATCACTTATATGTAGTATCTGTCAT | CACACCTGTTGCCCTTACTTGATT | 500 |
| SOX5_14 | chr12:23737233-23737768 | AACACTAGGGTGCTGCCTTAGG | AAATGAAATACTTTGAAGGACTCCCAGA | 536 |
| SOX5_15 | chr12:23728396-23728956 | ATGTATAGTTGAAGGAGGCATTATGTTGA | TTCAGGAGGGCATTATAACTTAGACTTTAG | 561 |
| SOX5_16 | chr12:23715999-23716504 | GGATCAGTCATTGTCAATTATTCATTCATTC | GAGAATTATAACTCTTTCTATTTCCAGATGGTT | 506 |
| SOX5_17A | chr12:23699067-23699590 | CCAAACCCAAACGCCAAGACTTTATAGG | GAGACGATTTCTGCAAAGCACTCAGC | 525 |
| SOX5_18 | chr12:23695975-23696499 | TGCCACCACAAGGCTTATCAGA | GGACATAGAATGCTTCATCACCAGAA | 525 |
| SOX5_19 | chr12:23689233-23689772 | AACCACCACCACCTCTGTTCTT | TCAAGATCATGTGGGAGACTATTTGTC | 540 |
| SOX5_20 | chr12:23685152-23687512 | CACATCTAACTATTCACTTACCCACGAT | AATGAAATCTCTGACATGCAGGATATGA | 2,361 |
